# Understanding the Cardiovascular Fallout of E-cigarettes: A Comprehensive Review of the Literature

**DOI:** 10.7759/cureus.63489

**Published:** 2024-06-29

**Authors:** Devansh Chaturvedi, Hussein Attia Hussein Mahmoud, Ashley Isaac, Ragha Harshitha Atla, Juveria N Shakeel, Maria Heredia, Nitheesha Reddy Marepalli, Pranav S Shukla, Maira Gardezi, Madiha Zeeshan, Tehreem Ashraf

**Affiliations:** 1 Medicine, Dr Chaturvedi Cancer Hospital and Research Institute, Gorakhpur, IND; 2 Internal Medicine, King George's Medical University, Lucknow, IND; 3 Diagnostic Radiology, Heliopolis Hospital, Cairo, EGY; 4 General Medicine, Isra University Hospital, Hyderabad, PAK; 5 Internal Medicine and Obstetrics, Bicol Christian College of Medicine, Ago Medical Center, Legazpi City, PHL; 6 Medicine, Trinity Medical Sciences University, Kingstown, VCT; 7 Cardiology, Ministry of Public Health of Ecuador, Quito, ECU; 8 Internal Medicine, Dr. Patnam Mahender Reddy Institute of Medical Sciences, Hyderabad, IND; 9 Medicine, Grant Medical College and Sir JJ group of Hospitals, Mumbai, IND; 10 Internal Medicine, Faisalabad Medical University, Faisalabad, PAK; 11 Internal Medicine, Mid Michigan College, Michigan, USA; 12 Medicine, Islam Medical College, Sialkot, PAK

**Keywords:** nicotine, pregnancy, e-cigarettes, particulate matter, cardiovascular complications

## Abstract

E-cigarettes (ECs) deliver chemicals, including nicotine. They can cause respiratory distress, addiction, cardiovascular effects, and death. More research is needed, especially regarding their impact on the cardiovascular system (CVS) and during pregnancy. Our article aims to fill this gap by summarizing studies elaborating upon the current impact of ECs and the components thereof on the CVS. Acute respiratory distress outbreaks, nicotine addiction, CVS effects, and deaths have been occasionally reported within this cohort, although these events are not uncommon with neighboring age groups. Randomized control trials implying ECs have some contribution toward quitting smoking have been studied. To regulate EC distribution, the Food and Drug Administration (FDA) and Centers for Disease Control and Prevention (CDC) have created key checkpoints. Additionally, taxation, pricing, age restriction, and media campaigns could be modulated to significantly reduce illicit sales. Education to the users, distributors, and regulators about this product can also play an aiding role in promoting responsible EC use. Another strategy about licensing could be employed, which could incentivize genuine resellers. The effects on CVS and child-bearing by ECs are grim, which calls for strict regulation, awareness, and avoidance by the teetotaler public. They may help individuals stop smoking but not without harming themselves. Strict regulations are necessary to prevent non-judicious use of these devices.

## Introduction and background

An e-cigarette (EC), also referred to as "JUUL" (brand name), electronic vape, e-pen, e-hookah, e-cig, or simply vape, can be defined as a device used to consume aerosolized chemicals, usually containing nicotine and other base constituents (flavorings, propylene glycol, vegetable glycerin, and similar additives) by vaporizing and inhaling its fluid equivalent, colloquially referred to as "e-juice or an 'e-liquid" [1,2]. Though these devices may or may not contain nicotine in their fluid, they are collectively called electronic nicotine delivery systems (ENDS) [3,4]. They may be disposable or refillable tanks [5]. "E-concentrates," another product purchased frequently with ENDS, are refined solutions with elevated levels of propylene glycol or flavors that can be diluted further to make an "e-liquid" according to a user's preference [6].

Some articles refer to ECs in terms of "generations": the first generation is called "cigalikes" or "disposable e-cigs" due to their uncanny resemblance to traditional tobacco cigarettes (TTC), their "once-only" use (attributable to a single battery charge), and their disposable nature. The second-generation ECs are designed for multiple uses and feature cartridges that can be refilled with an e-liquid of choice as often as needed [5]. The third-generation EC builds upon the customizability of this device. Sub-ohm tank, for instance, is a third-generation EC that specializes in generating high aerosol output (or "cloud"). They are informally called "mods," referring to their hyper-customizable nature. Similarly, "pod-mods" is the informal term for fourth-generation ECs, constituting a multi-use "pod" and a hyper-specialized delivery system (the "mod"). "JUUL" belongs to this generation [1].

"Vaping" or "Juuling" is the term for nicotine-based EC consumption, while when used with cannabis, a blanket term called "dabbing" is used for the act. The cannabis-based EC is then referred to as a "dab-pen." "Hacking" the "mod" refers to the user-made modifications to the EC system that the manufacturer unintended [7]. The gadget was initially conceptualized as a means to deliver medicinal components, and throughout its evolution, it has displayed much potential for aiding TTC consumers to quit smoking; however, it is debatable the extent to which it can achieve this even today, owing to regional and logistic variations explicitly about the generation, method, setting, and country of EC consumption [8]. Notably, the US and the UK have clashing views on this. The degree of strictness and structured approach concerning ENDS distribution underpins these erratic states of affairs [9].

More than 460 different EC brands are currently on the market [10]. Despite numerous differences in appearance and design, many of them have a similar operation concept. The average EC/vape consists of four primary components. Namely, a cartridge/reservoir/pod, usually containing a liquid solution (e-liquid or e-juice) with various flavorings and chemicals, a heating element (atomizer), a power source (usually a battery), and a mouthpiece that the person uses to inhale. Upon activation of the heating coil, the cartridge solution is vaporized before being inhaled by the user. This activation is usually initiated by puffing [10].

## Review

Types of electronic nicotine delivery systems

**Figure 1:**
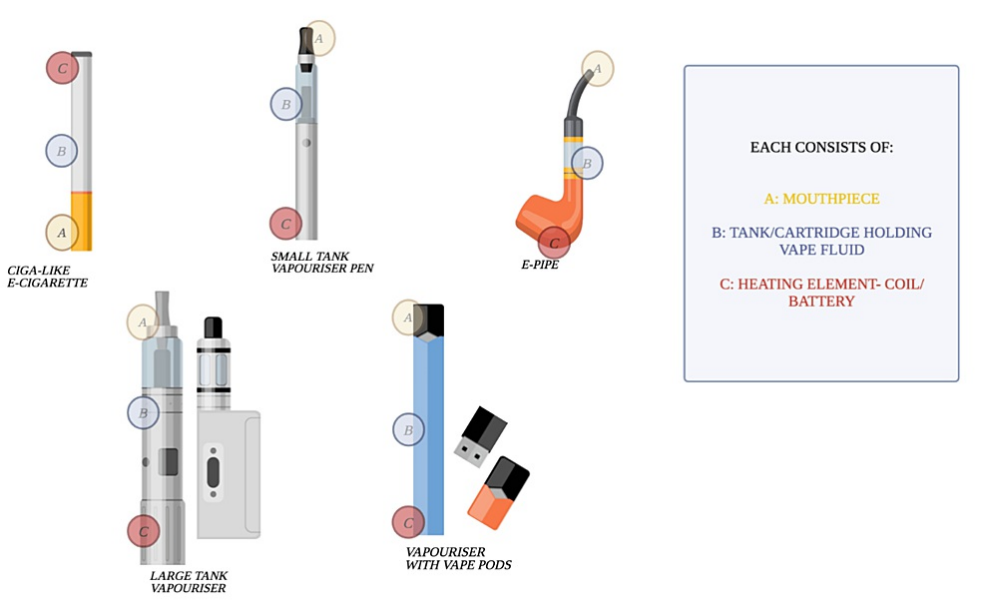
Various electronic devices for the delivery of nicotine

Figure 1 below shows some electronic devices for nicotine delivery. Electronic nicotine delivery systems (ENDS) devices are a broad category of battery-powered gadgets that emit an aerosol a user inhales that contains nicotine and additional ingredients [11]. They come in various forms, but the most often used in the US are "pod"-based models that use liquids with a high nicotine content [12]. ECs can be classified on how they look.

Ciga-Like E-cigarettes

These are conventional tobacco cigarettes with a rechargeable battery and replaceable cartridges. When the person inhales this EC, a light is shown at the end of the battery, making it like a TTC. The working mechanism is that the liquid in the cartridge gets heated and is converted to vapors as the person inhales the EC.

Pod Vapes/Kits

Pod vapes are different from Ciga-like ECs in that they resemble a pen with a replaceable pod instead of a cartridge and a rechargeable battery. The pod consists of a coil mouthpiece and a tank filled with the e-liquid available in different flavors and other nicotine strengths [13]. Moreover, the pod vape's battery is long-lasting compared to ciga-like ECs.

Vape Pens

Vape pens, as the name implies, are pen-shaped and popular among vapers. Such ENDS have a similar construction to pod vapes, but the difference is that the coils of the vape pens have replaceable mouthpieces that can also be removed. However, like pod vape, the e-liquid comes in different flavors and is to be filled by the user. Vape pens have a button to be pressed to turn them on and cannot be used just by inhaling from the mouthpiece. This makes them different from pod vapes, where the user can turn the device on by inhaling it [14].

Box Kits

Box kits are advanced compared to the ENDS but are also heavy compared to pod vapes and vape pens. Box kits are very much like pod vapes and vape pens as they also contain a tank, coils, and a battery, but the battery here comes in a rectangular shape, and the box kits are designed in such a way that the tank protrudes from the leading case of the device.

Other classifications

Disposable Electronic Nicotine Delivery Systems

This gadget is meant to be used just once before being thrown away when the battery runs out, the e-liquid runs out or the atomizer wears out [11].

Non-refillable Cartridge Electronic Nicotine Delivery Systems

The user swaps the empty cartridge with a different disposable, pre-filled one [11].

Refillable Tank System Electronic Nicotine Delivery Systems

They are available in pen or box shapes, such as vape pens and tank pens, and some even feature digital screens [11].

Refillable Mod System Electronic Nicotine Delivery Systems

**Figure 2:**
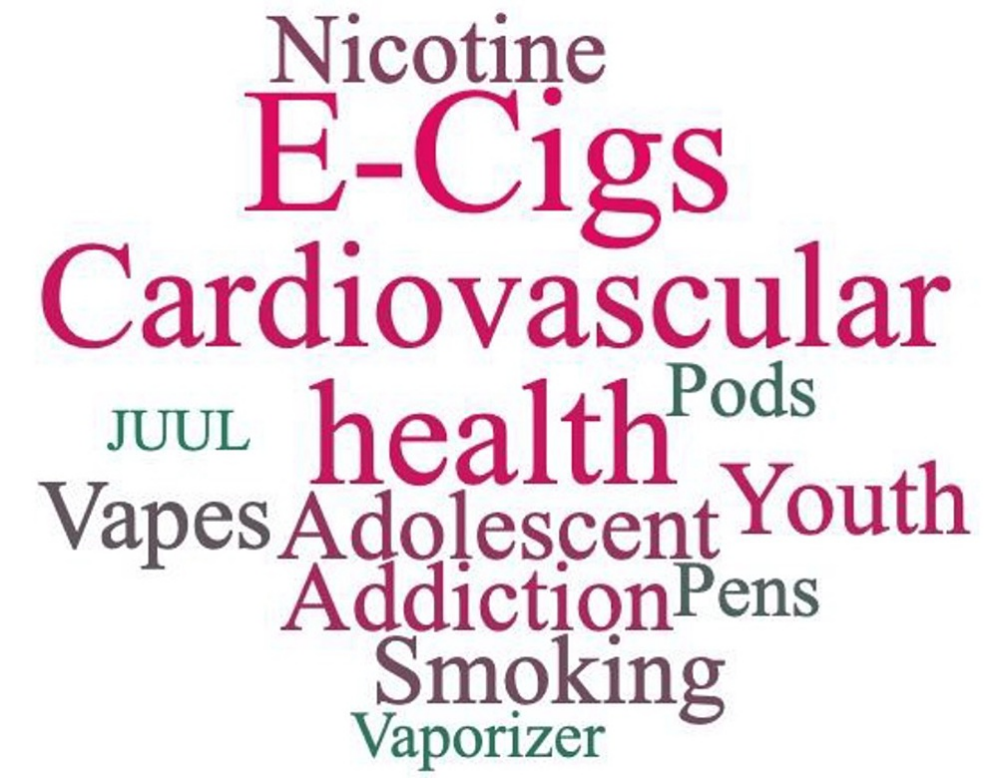
Word cloud showcasing different ENDS terms ENDS, electronic nicotine delivery systems

The users customize such devices. Like TTCs, EC users are exposed to chemicals and particles that impact several biological systems, such as the cardiopulmonary system [15]. Most ECs release and contain extremely varied chemicals that may be hazardous to cardiovascular health. Figure 2 shows a word cloud showcasing different ENDS terms.

Prevalence of e-cigarettes

**Figure 3:**
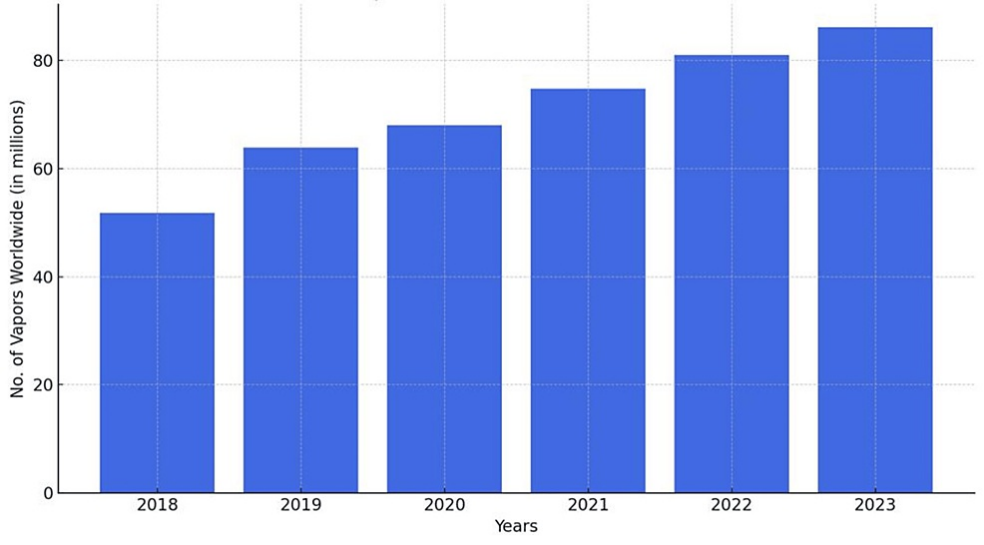
Number of vapors consumed worldwide from 2018 to 2023

ECs have evolved throughout time and across different regions. These were first introduced as a less harmful alternative from around 2007 onward. Nicotine is one of the world's most popular drugs, and about 68% consume it by smoking TTCs [16]. The goal of harm reduction advocates was to divert people from smoking TTCs in which users become addicted to nicotine and find it difficult to quit smoking. According to the WHO (World Health Organization), there were around 1.1 billion tobacco smokers in 2000, expected to remain unchanged until 2025 [16]. Survey data on global EC consumption and vape market users indicated robust growth, and this upscaling trend is expected to stay accelerated. Research from the Harm Reduction Journal estimated a linear trend in the global number of EC users in 2018-2023 (Figure 3). This estimation was based on data from 49 countries, and the missing data was interpreted by assuming similarities between countries with comparable attributes [16]. Figure 3 shows the number of vapors consumed worldwide from 2018 to 2023.

Figure 3 underscores the fact that EC use is not distributed evenly worldwide. Moreover, most EC users are situated in high-income countries. Several variables may contribute to this, including increased purchasing power and easier access to EC products. Moreover, in high-income countries, vaping products are marketed as a smoking cessation aid and used by smokers to quit TTCs [16]. North America is a leading market both from a global and regional perspective. The statistics from the Machine Learning Approach conducted among youth in California, where the market for e-cigs and vapes is forecast to grow at an estimated 38.9% from 2017 to 2030. Moreover, the statistics from the NCBI 2021 showed a rise to 43.6%, reflecting a concerning trend among the American youth developing dependence [17]. According to the National Health Interview Survey, there was increased use of ECs among Asian, Black, White, and Hispanic ethnicities from 2014 to 2020, particularly among adolescents. The aggressive marketing campaigns showing ECs as a less harmful alternative to TTC, the appeal of flavored e-liquids such as fruit and mint flavors, the growth of retail outlets, and the increasing trend toward internet shopping among young users likely played a role in this growing trend. A study of 30456 participants found that the weightage prevalence of ECs decreased from 5.2% in 2014 to 3.1% in 2019 but then increased again to 5.2% in 2020. It was found that youngsters and women more predominantly use ECs. The use of ECs among cardiovascular disease (CVD) patients decreased from 2014 to 2019, attributed to the reported harms of ECs. However, EC use rebounded in 2020, with more men smokers than women. This rebound may be explained by increased psychological burden during the COVID-19 pandemic [1]. According to a cross-sectional study, insufficient nutrition and substance misuse are the principal drivers of enhanced consumption [17].

Data from the Centers for Disease Control and Prevention (CDC) showed that in 2019, 36.9% of adults who used ECs also smoked TTCs, 39.5% had previously smoked cigarettes, and 23.6% had never smoked cigarettes. In 2021, 4.5% of adults in the United States used ECs. By 2022, 17.4% of middle and high school students in the United States had started using ECs.

Morbidity and mortality rates across the globe due to E-cigarettes

The global morbidity and mortality associated with ECs is a complex and evolving subject.

Morbidity

In 2019, a sudden outbreak of severe lung injury cases was reported in the US, primarily among adolescents and young adults [18]. These cases were associated with the usage of ECs or vaping products, and the condition became known as EC or Vaping Product Use-Associated Lung Injury (EVALI). Symptoms included cough, shortness of breath, chest pain, and sometimes gastrointestinal symptoms like nausea, vomiting, or diarrhea. The exact cause of EVALI is multifactorial, but vitamin E acetate, an additive in some tetrahydrocannabinol (THC)-containing EC products, was strongly linked to the outbreak [18]. Nicotine addiction among young users can harm brain development and increase the chances of other addictions [18]. Some studies suggest that EC use can adversely affect the respiratory and cardiovascular systems (CVS) [18].

Mortality

By the end of 2019 and into early 2020, several deaths in the US were attributed to EVALI [18]. The CDC reported that as of February 2020, there were 2807 hospitalized cases of EVALI from all 50 states, the District of Columbia, and two US territories (Puerto Rico and US Virgin Islands), out of which 66% were males with a median age of 24 years. Sixty-eight deaths were confirmed in 29 states and the District of Columbia by the CDC [18]. Beyond EVALI, determining the long-term mortality risks associated with EC use is challenging due to the relatively recent introduction of these products. It is also difficult to differentiate deaths related to EC use from those associated with traditional cigarette use since many EC users are smokers or former smokers [18].

Hospitalization

According to cases reported to the CDC as of January 7, 2020, 2558 patients were hospitalized, including fatal and nonfatal cases, among which there were 60 fatal EVALI cases. Among 60 EVALI cases, 32 were male. The proportion of patients with terminal or nonfatal cases was higher among those who were non-Hispanic white (39 of 49 (80%) and 1104 of 1818 (61%), respectively) than among those in other races or ethnic groups [18]. The proportion of fatal cases was highest among the age group of 35 and older. Among the patients whose medical history is available, fatal cases are highest among those with asthma, obesity, other mental health conditions, and cardiac conditions [18].

Toxic constituents in E-cigarettes and their harmful effects

Nicotine

Nicotine is an alkaloid; in its purest form, it is a clear, odorless, and colorless liquid. When exposed to air or light, it turns brown and gives off the pungent smell characteristic of tobacco. It is a chemical substance found in tobacco that is highly addictive, making it harder to quit smoking. The pleasurable feeling only lasts temporarily, which makes one reach for another cigarette in hopes of making the effects last longer. There are innumerable side effects to it that most smoking companies try to hide. Tobacco is the leading cause of preventable cancers worldwide. WHO estimates there are about 1.27 billion smokers worldwide. Tobacco use alone causes nearly 5.4 million deaths each year, and one billion people could die this century if global tobacco consumption remains at current levels [19].

Smoking is not just the leading cause of cancer in the developed and developing world but also the reason behind the rise in CVD. Tobacco smoke is prothrombotic and atherogenic, increasing the risks of acute myocardial infarction (MI), sudden cardiac death, stroke, aortic aneurysm, and peripheral vascular diseases [20]. The effects of nicotine on the CVS reflect the activity of nicotine receptors present in central and peripheral autonomic ganglia. Constriction of porcine coronary arteries induced by cigarette smoke extract is found to be associated with superoxide anion-mediated nitric oxide (NO) degradation [21]. Nicotine use results in acute hemodynamic effects, primarily via sympathomimetic action. This occurs by releasing local and peripheral catecholamine, leading to an increase in pulse rate, blood pressure, and cardiac contractility; the outcome is decreased blood flow in the coronary and cutaneous blood vessels but increased blood flow in the musculoskeletal system [22]. Even chewing a low dose of nicotine gum (i.e., 4 mg) daily by a non-smoker raises the coronary blood flow and heart rate, usually found in cardiac pacing. This can ultimately contribute to CVD by inducing acute myocardial ischemia; if a person has an ongoing coronary disorder, myocardial dysfunction exacerbates [22].

Nicotine modifies the structural and functional properties of endothelial and vascular smooth muscle cells. This intensifies the production of fibroblast growth factor and blunts the release of transforming growth factor beta 1, resulting in increased DNA synthesis, mitogenic activity, and endothelial proliferation. Nicotine use can initiate neovascularization, which can further advance atherosclerotic plaque formation [22]. This precedes myointimal thickening, atherogenic and ischemic changes, and increases the prevalence of CVD. The role of nicotinic acetylcholine receptors in the proliferation of vascular smooth muscle walls and in neovascularization also increases the frequency of peripheral arterial disorders; therefore, long-term exposure to nicotine (i.e., >4 months) will gradually prompt an angiogenic response [22]. The international treaty, introduced by the WHO in 2003 and signed by 170 countries, aims to encourage governments to reduce tobacco products' production, sale, distribution, advertising, and promotion. Despite strong industry opposition, the agreement steadily progresses toward comprehensive global tobacco control [22]. This is why alternative measures are sought to improve smoking cessation, such as nicotine patches, gums, inhalers, or even ECs. These products are available in most parts of the world and are marketed as incredible advancements. As an alternative to regular smoking, they contain much lower percentages but are still known to deliver at least 1 to 3 mg of nicotine into the bloodstream [22].

ECs, complex yet sophisticated ENDS, deliver nicotine in a vapor form and closely simulate smoking. These products account for about 1% of total nicotine consumption and are gaining popularity at an alarming rate in most countries [22]. An EC is a battery-powered device that heats a liquid consisting of propylene glycol (PG) and vegetable glycerin (VG), nicotine, and flavoring to form an aerosol that is inhaled like a cigarette containing all these chemicals that are known to be carcinogenic [23]. The amounts of many of these chemicals appear lower than in traditional cigarettes. Still, the levels of nicotine and other substances in these products can vary widely because they are not constant. According to the American Journal of Respiratory and Critical Care Medicine (AJRCCM), the nicotine content in ECs typically ranges from 3 to 36 mg/mL. Newer ECs contain more nicotine (up to 60 mg/mL), often in salt form, to accelerate and increase nicotine delivery to brain levels comparable to traditional cigarettes [24].

Therefore, evaluating ECs' short- and long‐term safety on the CVS is vital, especially given the limited research in this area and their contentious findings. Many studies have suggested that ECs impact vital signs acutely and devastatingly, such as heart rate and blood pressure. In this aspect, Andrea et al. demonstrated that smokers' heart rates increased after EC use, which was also seen in a different study. Furthermore, Yan et al. found that e‐cigarettes increased diastolic blood pressure and heart rate in smokers but not as much as in tobacco cigarette smokers [25]. EC use causes a borderline and swift rise in circulating endothelial progenitor cells, possibly due to intense endothelial and vascular injury. The most recent study recommends that platelets significantly aggravate CVD, especially thrombosis and atherosclerosis. In this manner, EC vapor extrication improved platelet actuation (accumulation and grip) in many volunteers [26].

A study published in the American Health Journal in 2017 by D'Ruiz et al. concluded a rise in heart rate after using ECs concerning soaring plasma nicotine levels. The results were evident in EC users 5 minutes after the first puff and even after 1 hour [27]. Ancillary concerns about ECs are nicotine dependency and toxicity, given that the nicotine levels found in the plasma of EC smokers are high enough to initiate and sustain nicotine dependency and could explain why so many adolescent EC users find it so difficult to quit and carry on with the habit of vaping into their adulthood. The amount of nicotine in these ECs is also known to be fatal in young adults. Calls to poison control centers regarding e-liquid ingestion increased "from one call per month in September 2010 to 215 calls per month in February 2014." The law on preventing nicotine addiction in children came into force in January 2016. This has prompted EC manufacturers to offer safe e-liquid packaging for children [28].

Despite various studies still advancing the thought of choosing ECs as a more advantageous elective to tobacco smoking, it is crucial to calculate the health risks these may cause to non-users within the same region as the users. It can be inclined to use vaping/exposure. Additionally, another risk has come to light, that is, thirdhand smoking/vaping from the nicotine molecule buildups that still stay within the air and the surfaces hours after it was smoked upon. Different studies have demonstrated with significant proof that ECs are not nicotine‐free gadgets; on the contrary, they worsen indoor air quality and ultimately, in the long run, have the same harmful effect as traditional cigarettes [11].

Carbonyl

ECs emit considerable levels of carbonyls [29]. This group of compounds includes formaldehyde, acetaldehyde, and acrolein [30]. These are thermal degradation products of PG and glycerol, the most used solvents for EC liquids [31]. Formaldehyde is a genotoxic chemical mainly associated with nasopharyngeal carcinoma and myeloid leukemias [32,33]. Acetaldehyde irritates the upper airway mucosal surfaces with less clear systemic effects [34]. Acrolein is a highly water-soluble unsaturated aldehyde with systemic effects due to various pathways, including direct adduction of protein and DNA, oxidative stress, and mitochondrial dysfunction [35]. Potential cardiovascular effects of acrolein include dilated cardiomyopathy, natural cardiomyocyte apoptosis, and exacerbation of ischemic reperfusion injury [36-38].

Heavy Metals

There is much concern about the inhalation of heavy metals to which EC users are exposed. Heavy metals have demonstrated adverse implications in EC users, causing severe health problems, including CVD, kidney damage, neurotoxicity, and cancer [39]. The metals in the highest concentration in patients' serum are lead, arsenic, chromium, and nickel. It is known that chromium and nickel are known so far to be carcinogenic and neurotoxic [40].

ECs contain liquids derived from dangerous substances when they enter the combustion process, creating free radicals. It also could be seen when the device gets heated, and its soldered joints of heavy falls are expelled into the respiratory mucosa. All these factors worsen when the duration of vaping puff is more remarkable. When the liquids in the ECs are heated and converted into inhalable aerosols, they convert certain substances into degradation products such as formaldehyde, acetaldehyde, and acrolein, which has been used as a weapon and herbicide, generating free radicals that are harmful to cells, causing vascular oxidation with the consequent platelet activation becoming a risk factor for thrombosis. Acrolein also causes lipid peroxidation and conformational changes in high-density lipoproteins and Apo A-I, thus being the origin of the development of an atherosclerotic disease [41]. One of the most studied heavy metals is arsenic. A study found 27 ng/g of arsenic in the liquid ECs and aerosols, which does not exceed the US Environmental Protection Agency's National Ambient Air Quality Standards (NAAQS). However, chronic exposure to arsenic causes peripheral vascular disease, hypertension, and CVD [42]. The extent of cardiovascular damage may vary depending on age, arsenic dose, and individual susceptibility.

On the other hand, the chromium metal found in the welds of ECs has demonstrated its effect on the respiratory tract, causing neoplastic disease, dermatitis, ulcerative disease in the upper respiratory tract, and kidney damage [43]. Lead has been linked to higher levels than other metals in EC aerosols, and lead levels are likely increased in the blood of EC users [39]. This metal is thus a risk factor for cerebrovascular disease and coronary artery disease. The exposure of EC users to nickel has been associated with respiratory rather than cardiac problems due to the inflammatory changes produced in the respiratory mucosa. The most related diseases are sinusitis, bronchitis, and lung cancer [39].

Particulate Matter

EG-generated aerosol contains particulate matter with a diameter <2.5 microns (PM2.5). Particles of this size may injure the health of those inhaling them, leading to respiratory and CVDs. Many studies show indoor particulate levels during EC consumption to be >150 μg/m^3^, similar to TTCs. The effect of ECs on indoor levels of PM2.5 is nearly the same or sometimes higher than that of other non-combustion-free nicotine delivery systems. The size distribution and number of particles in the particulate matter (PM) e-vapors emitted by ECs vary depending on the e-liquid, nicotine concentration, and puffing topography [44]. ECs, also known as vape pens, contain a special liquid and a battery that helps heat the liquid into aerosols that the users inhale. This liquid that fills the cartridges contains nicotine, PG, flavorings, and other harmful chemicals. Studies have found that even ECs claiming to be nicotine-free contain trace amounts of nicotine [45]. Additionally, when the e-liquid heats up, more toxic chemicals are formed.

Effect of particulate matter from E-cigarettes on the cardiovascular system: Ultrafine and delicate PM (PM0.1, PM2.5) is found in significant concentrations in ECs [46,47]. It is known to elicit several cardiovascular effects and contribute to atherosclerosis, thrombosis, coronary heart disease, and hypertension. Mechanistically, these effects occur through both direct and indirect pathways. PM0.1 and PM2.5 can pass through the alveolar-endothelial interface into the systemic circulation, directly affecting the heart, vasculature, and other organs. The thrombogenic effects of PM are believed to arise from the promotion of clot formation after entering the systemic circulation and from pulmonary inflammation caused by prolonged exposure [48]. Specifically, the mechanisms by which PM directly induces cardiovascular effects are through oxidative stress and direct calcium ion channel interference of the autonomic nervous system (ANS), and pulmonary inflammation results in PM-induced indirect cardiovascular effects [49,50]. Additionally, PM2.5 has been shown to cause direct dysfunction of vascular endothelial cells, contributing to the development of CVDs, particularly atherosclerosis [51].

Clinical and epidemiological research point to a direct link between human exposure to PM2.5 and the risk of CVD. Such studies have specifically demonstrated a connection between hypertension and exposure to PM2.5 from background air pollution and conventional cigarette use. It has been speculated that PM impacts cells outside the lungs for many years. However, there is little proof that particles may enter the bloodstream and have such an impact. The first instance of this data came from Nemmar et al. (2001), who showed that ultrafine particles (UFPs) could enter both the pulmonary and systemic circulations [52]. This result was rapidly confirmed by Nemmar et al. in 2002 [53].

Mechanism of how particulate matter particles can affect the cardiovascular system: Delivery of PM2.5 into the bloodstream, which targets numerous organs, mediates the direct channel [54]. Thus, if ion channels and calcium regulation are affected by PM2.5, it could lead to contractile dysfunction and arrhythmia [54,55]. Regarding the indirect pathway, PM2.5‐induced cardiovascular toxicity is associated with developing inflammatory responses and modulation of the ANS dysfunction, and thrombus formation can result from producing local oxidative stress and inflammation [55-58]. Thus, the deposition of PM2.5 on alveoli triggers the release of a host of pro-inflammatory mediators, vasoactive molecules, and reactive oxygen species (ROS) into the circulation. These subsequently affect vascular integrity and induce thrombogenesis [57,58]. As for PM2.5, modulation of the ANS results in increased vasoconstriction and changes in heart rate variability, potentially enhancing the risk of developing arrhythmias and thrombosis [59].

Cardiovascular effects of e-cigarettes

Vaping is often limited to the afflictions of the respiratory tract; however, fractions of unmetabolized e-vapor often make their way to the CVS, which may exhibit genotoxic or mutagenic effects. E-liquid flavor varieties are the major driving factor for adolescent appeal and consequent abuse. Recent evidence surfaces on the harmful effects of these chemicals inducing cytotoxicity. Notable flavors with this effect include cinnamon and cookies, berry and herbal (B&H), butterscotch, and menthol and cinnamon (M&C). Among the in vitro flavor studies, B&H was implicated with prominently staggered proliferation, increased morphological alterations, and monolayer disruption in human umbilical vein endothelial cells (EnC) [60].

Conversely, cinnamaldehyde among "e-concentrates" was also considered a contributor toward disrupting EnC integrity in human induced pluripotent stem cell (hiPSC)-derived cardiac myocytes. In human aortic epithelial cell tests, a subset of tested flavors (vanillin, menthol, cinnamaldehyde, eugenol, dimethyl pyrazine, diacetyl, isoamyl acetate, eucalyptol, and acetyl pyrazine) impaired NO production, possibly via ROS scavenging of NO [60]. They also upregulated interleukin-6 (a proinflammatory cytokine) levels. M&C plummeted hiPSC EnC viability via increased caspase 3 and 7 activity and shortened tube formation, affecting angiogenesis. Cinnamon and caramel/vanilla increased low-density lipoproteins and free fatty acids uptake by these cells. A similar study on 34 e-concentrate and 21 e-liquid flavors suggested varied forms of demonstrable cytotoxic effects, with cinnamaldehyde leading the pack [60].

On the other hand, in vivo studies on rats incorporated exposure to aerosols from JUUL, 3rd, 2nd, and 1st generation ECs. All exposures were associated with EnC dysfunction to some extent, as indicated by changes in arterial dilation. The choice of flavor may affect nicotine uptake and absorption, which indirectly has varied cardiovascular effects. However, research on this aspect would be challenging due to the many available flavors [60]. These findings indicate a more prominent role of the choice of vapor constituent and the generation of EC toward cardiovascular detriment, of which the 2nd generation devices were the most commonly associated. Some flavors purportedly affected myocardial fibroblasts at par with regular TTCs. These concerns led to the selective banning of specific EC generations by the US Food and Drug Administration (FDA) on Jan 2, 2020 [60].

An independent in-vitro study in 2023 on RAW264.7 cell lines, which are considered the prototype macrophage cells for testing immunological impacts of novel chemicals, confirmed decreased phagocytic activity and increased levels of ROS upon exposure to EC vapor wherein the aerosol influx was calibrated to 10 puff/hour dose over 48 hours, simulating typical habits of an average consumer [61]. Excessively high ROS levels correlate to cell senescence, a risk factor for hypertension. Defective phagocytosis links to a weakened immune system, a driving factor for chronic illness offset by some of the weak pathogens. A higher probability of organ fibrosis is associated with EC aerosol exposure owing to findings of elevated key cytokines (interleukin-6, tumor necrosis factor-alpha, and interferon-beta). Although ECs proved less toxic than TTC in this study, neither proved better than not consuming them in the first place [61]. Nicotine and its cardiovascular effects are complex. Acutely grim manifestations do not necessarily translate into more severe forms over long-duration exposures. Adverse outcomes frequently manifest only when other components are thrown in the mix, hinting that short-term derangement in cardiovascular parameters is an expected finding, but comparative 10-year studies that definitively point toward nicotine as a toxic component are lacking and are worth exploring. Most studies have smoking (EC or TTC) as a confounding factor with nicotine exposure [62].

A randomized, double-masked crossover clinical trial propounded specifically nicotine-based formulations to increase platelet aggregation and thrombosis. Nicotine-driven catecholamine surge reportedly causes spikes in blood thrombogenicity and blood pressure with a simultaneous drop in peripheral perfusion. This phenomenon appears dose-dependent but is also positively connotated with the delivery method used: bolus systems similar to vaping show maximum thrombotic effect, while transdermal patches are the least. Nevertheless, nicotine as an EC component may contribute significantly to cardiac events [63].

Additionally, Feng et al. demonstrated accelerated renal fibrosis upon nicotine-containing EC aerosol exposure to mice feeding on a long-term high-fat diet, spiking alpha-smooth muscle actin (alpha-SMA) levels known to cause myofibroblast proliferation. Fibrosis observed in the control group was significantly lesser. A magnifying effect of nicotine EC on pre-existing oxidative stress was implied, resulting in renal fibrosis [64]. Additionally, increased atherosclerosis and modified gene expression, primarily upon nicotine exposure during developmental age, have also been reported, indicating the need to check for confounding additives and potential nicotine interactions within ECs [65,66].

There seems to be a particular psychological archetype that especially resonates with ENDS usage, which is associated with unintended but inevitable cardiovascular states. A higher perception of stress, multiple cessation failures, and a tendency for individuals with this psychological profile to co-consume it with TTC potentially lead to a higher risk of developing metabolic syndrome. Nicotine-dependent weight accumulation and confounding factors of long-term depression go hand in hand with such personality types to build these cardiovascular outcomes [67]. Similarly, a prevalence study by Alzahrani et al. in a local setting also indicated a higher probability of such users being obese, drug addicts, and male, with the most commonly reported reason for ENDS consumption allegedly being recreation and mitigating depression. The same users also coincidently self-reported experiencing palpitations and chest pain [68]. Interestingly, a recent study shows that EC users are more likely to suffer from depression, suicide ideation, and suicide attempts [69].

ENDS use is associated with an increased risk of MI and the potential to exacerbate pre-existing cardiopulmonary diseases [70]. Adult EC users who also smoke cigarettes are known as dual users; in the United States, 39.1% of users reported using ECs in 2018-2019; in Sweden, 66.7% in 2016; and in Korea, 85.3% in 2013-2017 [71]. Any form of cigarette consumption, traditional or otherwise, is associated with an increased pulse wave velocity (PWV), an indicator of arterial stiffness. Rapid generation of nicotinamide adenine dinucleotide phosphate oxidase (s-NOX2-dp) mediated ROS, infamous for causing vascular dysfunction, may result from nicotine or other constituents (especially acrolein) within the vape. Compared to tobacco-flavored ECs, menthol-containing EC aerosols have higher concentrations of formaldehyde and acrolein, which makes them more likely to cause ventricular premature beats [72]. Nicotine, in particular, is known for its sympathomimetic effects, left ventricle (LV) remodeling, and potential rhythm disturbances of the heart. Although human heart tissue studies were not mentioned, it is worth noting that the toxic cardiovascular effects of ECs were known in mice over three to six months of chronic EC exposure above a specific dose. It suggests a time lag between starting EC consumption and witnessing outcomes concerning cardiac myoblasts [73]. The total odds ratio for sickness associated with all EC use in a population is increased when one takes into account the fact that some users only use ECs and others use them in addition to other tobacco products. This is because dual use is linked to odds ratios over one for all outcomes. The probability of odds ratios larger than one are 0.42 for cardiovascular illness, 0.28 for stroke, greater than 0.99 for metabolic disorder, 0.30 for asthma, 0.98 for COPD, and 0.77 for oral disease, based on 39.1% of dual users in the United States in 2019 [71].

Gordon et al. in early 2022 indicated that these modified cigarettes indeed acutely cause derangement in blood pressure, heart rate, sympathetic dominance, increased endothelial progenitor cell numbers, increased bioavailability of iso-prostaglandin F2α and FeNO, increased levels of vitamin E, greater activation of platelets, reduced flow-mediated dilation and higher arterial stiffness. Moreover, in certain mice studies, atherosclerotic plaque development was accelerated. Although occasional comparisons with TTCs were made, it was evident that these newer methods of cigarette use were less, albeit definitively, damaging in comparison. No such dynamics were observed in a minimal subset of cases, subtly proposing whether ECs were detrimental. Interestingly, attempts at quitting TTC addiction using ENDS may be associated with regression to dual dependence on both conventional and ECs [74].

Different studies indicate exclusive ENDS consumption, compared to TTCs, is associated with a "less damaging" risk profile [75,76]. A drop in thirteen biomarkers (12 renal, one hematological) was reported upon switching to ECs, implicating better cardiovascular parameters for individuals switching from TC to EC [77]. However, Esteban et al. in late 2022 pointed to definitive studies on human cell culture lines suggesting equal, if not greater, harm done by both cigarette types. Specific reference to staggeringly high levels of nicotine delivery by specific ECs (JUUL: 136.4 ng/mL, other EC: 17.1 ng/mL) than TC (26.1 ng/mL) was emphasized as a mediator to both cerebral and cardiac consequences, including aortic aneurysms, followed by acrolein aldehyde, which promoted NADPH-oxidase (NOX2) mediated damage [78].

A case was reported linking ENDS usage to thrombotic events as an indirect consequence of EC or EVALI [79]. Balinsky et al. reported central retinal vein occlusion (CRVO) in a young patient with a four-year vaping history [80]. Possible pro-thrombotic interactions with oral contraceptive pills (OCPs) were implied after excluding organic causes for a 22-year-old woman presenting with a chief complaint of recurrent seizures [81]. Two case reports also suggested possible vaping-associated cardiomyopathy. Both reported an acute drop in LV ejection fraction to around 32% (previously expected), a dilated LV, and diffuse hypokinesis. The 35-year-old bodybuilder male had a previous cardiac history of aortic pseudoaneurysm and partial aortic resection with subsequent transthoracic aortic repair. The 19-year-old had a history of recreational oral opioid and benzodiazepine use. Although these patients' conditions deteriorated suddenly, their recovery was rapid due to radical therapeutic interventions. This predicament was reported to be probably caused by the nicotine in vape, as it is known for causing cardiac sympathetic stress, which suggests a tendency of adverse cardiovascular events to precipitate as a cascade in a particular cohort of individuals with pre-existing deranged cardiovascular profiles preemptively influenced by lifestyle choices [82,83].

A meta-analysis in August 2023 comprising 1024401 subjects confirmed increased stroke risk of roughly >50% in active ENDS users in comparison to non-users, purportedly due to collective effects of blood-brain barrier damage, blood pressure elevation, vascular repair impairment, endothelial dysfunction, and platelet activation. Since no such association was obtained for people who were not actively vaping, it was emphasized that smoking cessation is a linchpin for preventing stroke [84]. Concerning toxicity from individual components, the American Heart Association (AHA) implicated cardiac remodeling and arrhythmogenesis as a long-term consequence of nicotine use. It can also increase stroke and ischemic brain injury risk. Sepsis-like states owing to acute kidney injury and metabolic acidosis were attributed to PG as well as thrombosis and dyslipidemia [72,83]. Flavors were implicated with varying effects on cell lines within an inflammatory umbrella: menthol caused low cell viability; RY-4 e-liquid caused greater macrophage activation; vanillin, menthol, and eugenol impaired NO production; and cinnamaldehyde was involved in almost all of these. This leads to cytotoxicity due to oxidative stress [72]. Acrolein in nicotine-free vapes has been shown to disrupt endothelial barrier functioning [85].

Another frequently abused chemical, cannabis, can be used as an extract within the e-liquid or inhaled separately in the conventional way. Vaped cannabis, typically shipped with greater THC than cannabidiol (CBD), shows higher bioavailability and, consequently, more significant psychological effects when consumed in this manner [86]. It also interacts with nicotine absorption, which could ultimately modulate cardiovascular effects. It is not unusual for companies and distributors to mix synthetic cannabinoids with typical cannabis vapes, which would pose a significant disaster for the user, as the former is associated with higher morbidity and mortality [86].

From a biological perspective, THC-dominant marijuana, often infamously adulterated with synthetic cannabinoid derivatives, increases the risk of adverse cardiovascular events when smoked traditionally. The risk of ST elevated MI is reported to increase to 500% within an hour of consumption in healthy subjects without pre-existing comorbidities [87]. This hazard is collectively attributed to a blunted sympathetic drive, dysfunctional endovascular and endothelial behaviors, accelerated atherosclerosis, and pro-coagulatory effects on platelets, culminating in coronary vasospasm within the first hour of ingestion. These mechanisms also extend to causing both ischemic and hemorrhagic stroke, with the latter explicitly seen with synthetic derivatives [87]. Cannabis can be a silent promoter of Beurger's disease through cannabis arteritis, especially with chronic tobacco abusers who are likely to consume both together. Unchecked abuse can potentially lead to limb amputation [87].

Interestingly, these effects seem to be mitigated, not absent, in CBD-dominant variants, which the lack of conclusive epidemiological evidence for clear causation and correlation between cannabis and cardiovascular accidents can support. Unfortunately, it is entirely random which variant an adult consumes [87]. The effects of cannabis in e-liquid format are not extensively researched, and we can only speculate that a vaped cannabis format would pose a modulated impact on the CVS.

Ergo, it is now well established that ECs pose severe cardiovascular risks, whether used alone or in combination with other plant-based drugs. It should not be consumed in any form for any reason whatsoever. When used alone, it may help in cigarette cessation but does not cure depression; it thrives on psychological dependence and co-morbid helplessness, stopping only with near-death or debilitating cardiovascular events. As business and marketing strategies are constantly evolving, it would not be surprising to have a rebranded product in the future with a similar side effect profile, countered only by the ones with a curious and vigilant mind.

E-cigarettes in pregnancy and their adverse outcomes

Over the past few years, ECs have been widely used, even among pregnant women. There is insufficient data regarding the correlation of nicotinic exposure before or during pregnancy. Yet, women in their child-bearing periods are strongly advised to avoid exposure, whether active or passive smokers [88]. Recent observational studies show that smoking would lead to several undesirable side effects in pregnancy that range from risks of low birth weight (LBW) and sudden unexplained death in infancy (SUDI) to problems during childhood, such as respiratory and non-communicable diseases [89].

In contrast, a meta-analysis study in 2011 related to congenital disabilities proved that maternal smoking is associated with a notable augmented risk for cardiovascular defects with an odds ratio (OD) of 1.09 and a 95% confidence interval (CI) of 1.02-1.17 [90]. Another study in 2013 concluded that pregnancy smoking increases congenital heart defects (CHD) with a relative risk (RR) of 1.11; 95% CI of 1.02-1.21 [89]. This study had an evident heterogeneity for CHDs, with a p<0.001. However, fixed effects such as double-outlet right ventricles (95% CI, 0.72-1.46; n cases=179) showed a positive association similar to random effects like septal defects (95% CI, 1.16-1.79; n cases=2977) [91]. The pooled RR of any CHD was 1.11 (95% CI: 1.04, 1.18; I2=69.0%, p<0.001) [92].

EC constituents are likely to cause a hypoxic fetal environment due to placental insufficiency [93]. In two different meta-analyses, Shobeiri et al. (2017) emphasized the fact that smoking during pregnancy leads to an increased risk of experiencing placental abruption (OR 1.80, 95% CI 1.75, 1.85 and RR ratio: 1.65, 95% CI 1.51-1.80) and increased risk of placenta previa (OR 1.42, 95% CI 1.30-1.54 and RR 1.27, 95% CI: 1.18-1.35) in comparison to non-smoking pregnant patients [94,95]. A study in 2016 by Suliankatchi showed that smokeless tobacco by females in their child-bearing period doubles the risk of having LBW babies (OR =1.88, 95 % CI 1.38-2.54), preterm babies (OR = 1.39, 95 % CI 1.01, 1.91) or even stillbirth (OR = 2.85. 95 % CI 1.62-5.01) [96].

Research showed pregnant females who used vapes, specifically flavors such as mint/methanol, had a higher risk of fetal death than other flavors. In contrast, other females who quit vaping before conceiving had no likelihood of having a risky pregnancy compared to non-smokers [97]. The usage of ECs has been a significant public health concern due to the misconception among the population who consider that ECs are potentially safer alternatives to tobacco smoking [97]. Therefore, it is recommended to start initiatives that support more research in finding the effective pharmacological intervention with the required dosage to help women in smoking cessation before conception. Additionally, more research with a long-term follow-up is needed for a better understanding of the behavioral circumstances of women during their child-bearing period [97].

Recommendations and future plans

Although conventional smoking has decreased significantly in recent decades among young adults in the U.S. Department of Health and Human Services (USDHHS) 2012), there has been a significant increase in the use of emerging tobacco products among this population in recent years according to the CDC 2015. Among these is a marginal increase in the use of ECs in young adults. Significantly, progress in reducing smoking among adolescents and young adults is not influenced by EC initiation and use [98].

Direct claims about the effectiveness of smoking cessation are prohibited by law. Still, it is easy for EC manufacturers to make indirect claims about smoking cessation through testimonials from product users. Case reports and one small prospective study have suggested the potential effectiveness of ECs as a smoking cessation aid [99]. Only two large randomized clinical trials evaluating the efficacy of ECs for smoking cessation and reduction have been published. In a 12-month randomized clinical trial of 300 cigarette smokers not intending to quit tobacco, participants were randomized to one of three EC groups: Group 1: a 12-week supply of 7.2 mg EC nicotine cartridges; Group 2: a six-week supply of 7.2 mg EC nicotine cartridges and then a six-week supply of 5.4 mg EC nicotine cartridges; and Group 3: a 12-week supply of cartridges that contained no nicotine. Decreased cigarettes smoked per day and exhaled carbon monoxide levels were observed across all three groups. At the end of the treatment period, the percentage reduction in cigarettes per day was 26%, 20%, and 21% for the three groups, respectively. Tobacco abstinence rates at 12 weeks were 11%, 17%, and 4%, respectively (P=0.04). However, no significant differences were observed between the groups at six and 12 months. Another clinical trial randomized 657 smokers who wanted to quit to a 16 mg nicotine EC, a 21 mg nicotine patch, or a placebo EC [100]. Low-intensity behavioral support was provided through voluntary consultation over the phone. Smoking cessation was confirmed by measuring exhaled carbon monoxide levels. After six months, biochemically verified smoking abstinence rates were 7.3% with nicotine ECs, 5.8% with nicotine patches, and 4.1% with placebo ECs. No significant differences were observed in the study groups. Notably, this study's dropout rate was comparable to that observed in studies of over-the-counter nicotine replacement therapy when behavioral support was minimal or not provided [100].

Many practical strategies have been proposed to prevent tobacco use among young adults (USDHHS 2012), and many of these strategies can also be applied to ECs. A strategy to combat use among youth must be cautious. The precautionary approach encourages action to prevent harm in the event of scientific uncertainty. In other words, when knowledge is incomplete or premature, public health decisions must be made based on precautions aimed at preventing harm, not based on risk. This approach requires evidence that the product is not harmful, especially to young people, rather than evidence that the product is dangerous. The burden of proof of product safety will fall on those who want to market and sell these tobacco products rather than on the public health community responsible for protecting public health. Exercise the authority of the US FDA to regulate tobacco products to oversee the manufacturing, distribution, and marketing of ECs, especially as they relate to young adults [101]. Finance comprehensive statewide tobacco control programs at levels prescribed by the CDC [101]. Actualize comprehensive clean indoor air policies that secure individuals from exposure to used tobacco smoke and the vapors radiated from ECs [101]. Raise and strictly enforce the minimum sales age for all tobacco products, including ECs, to prevent young people from starting to sell them [101]. Establish EC pricing policies, including taxation [101]. Restrict advertising and marketing that encourages young adults to use ECs [101]. Sponsor effective media campaigns to educate the public on evidence-based information about the effects of EC use in adolescents and young adults, including the harms of nicotine to the developing brain [101]. Expand tobacco control and prevention research efforts to improve understanding of the evolving EC landscape [101].

Educate youth influencers about the risks of E-cigarette use among adolescents

Parents, teachers, and coaches can protect their children and other youth by raising awareness about ECs by talking openly about the harmful effects of nicotine and tobacco use and resolutely expressing that young people should not consume tobacco products, including ECs [101]. Do not let anyone use ECs or other tobacco products in front of children [101]. Sponsor restaurants and other places that do not allow indoor EC use and let business owners who do allow indoor EC use know that it is not as safe as clean air, even if it is legal in many places [101]. Ensure that nurseries, schools, and universities are completely tobacco-free, including ECs. The comprehensive tobacco-free campus policy prohibits anyone from consuming tobacco, including ECs, on school property at any time. These policies should include school events held off campus [101]. Prohibit tobacco and EC companies from sponsoring teams, events, and promotional activities [101]. All the recommendations mentioned above have become necessary since it has come to light that high schoolers are more likely to use ECs than adults.

Regulate E-cigarettes at the federal level to protect public health

In 2009, the Family Smoking Prevention and Tobacco Control Act (Tobacco Control Act) gave the FDA the authority to regulate tobacco products as "appropriate to protect the public health.” The Tobacco Control Act also requires the FDA to consider, in its regulatory actions, health effects at the individual and community levels, including the impact of initiating smoking cessation measures and the impact on relapse in people who have used tobacco [101]. A federal appellate court decision titled Sottera, Inc. v. The Food & Drug Administration (2010) has determined that the FDA may regulate ECs and other products manufactured or derived from tobacco under the Tobacco Control and Products Control Act that these products are not drugs or devices under the Food, Drug, and Cosmetic Act unless they are marketed as a smoking cessation or therapeutic product [101]. In May 2016, the FDA finalized a rule that most products that meet the definition of a tobacco product, including ECs, are subject to regulation under the Tobacco Control Act. This regulation took effect on August 8, 2016 (but is subject to litigation) (FDA 2016) [101].

The FDA's EC regulations include several provisions that can help protect teens and young adults from the harms of ECs, such as the prohibition of the sale of ECs to youth under 18 years of age (in person and online) and requiring proof of age at purchase [101]. Prohibit the sale of vending machines in all establishments where children are permitted and prohibit the distribution of free samples [101]. Require health warnings about nicotine on packaging and in advertising [101]. Require manufacturers to register their EC products with the FDA and disclose to that agency the composition and levels of harmful and potentially harmful ingredients in those products [101]. Before being marketed, require a premarket review of new or improved tobacco products and FDA authorization [101]. Require manufacturers intending to market ECs to reduce tobacco-related harm or disease risk to receive FDA authorization based on scientific evidence that the product causes more harm or poses less risk to the public [101]. Regulate packaging, including requiring minimum package sizes [101].

Licensing retailers

Licensing is another strategy to control access to ECs. Tobacco licenses can authorize a business to manufacture, distribute, or sell tobacco products (McLaughlin 2010) [101]. Licensing requirements help prevent the sale of tobacco to minors, prevent evasion of tobacco excise taxes, and ensure that licensees comply with tobacco laws and manufacturing practices (ChangeLab Solutions 2012) [101]. Businesses that repeatedly violate these laws may have their business rights suspended or their licenses revoked permanently. The possibility of such outcomes provides a solid incentive to comply with licensing requirements. Licenses may also be used to restrict the sale of flavored products or to address consumer and worker safety concerns related to mixing liquids for tobacco products (e-liquid) [101].

## Conclusions

The findings of our paper establish that the long-term use of ECs worsens cardiovascular health, providing strong evidence that ECs have a negative relationship with the CVS. Even though ECs may encourage smoking cessation, they are not related to a diminishment in nicotine use and dependency. They may lead to double utilization of ECs and TTC. Several relative harm assessment studies suggest that ECs cause less harm than smoking. However, not all outcomes measured (e.g., blood pressure) found harm reduction, and the extent of harm reduction when smokers switch to ECs is uncertain and requires further research. Moreover, there are distinctive discernments among young and adult EC users. The former group may utilize them because of the sense of fashion related to this gadget, and the latter might propose to quit conventional cigarettes by exchanging them for ECs. Be that as it may, it is vital to note that ECs are a later marvel; in this manner, there is a need for numerous long-term studies that can distinguish future well-being and dangers related to EC use, especially cardiovascular health. Additionally, with the ever-increasing utilization of ECs by teens, ECs must be consolidated into the current tobacco-free laws. We conclude by expressing that ECs require stronger directions to utilize.
